# Carbonate Mineral Formation under the Influence of Limestone-Colonizing Actinobacteria: Morphology and Polymorphism

**DOI:** 10.3389/fmicb.2016.00366

**Published:** 2016-03-23

**Authors:** Chengliang Cao, Jihong Jiang, Henry Sun, Ying Huang, Faxiang Tao, Bin Lian

**Affiliations:** ^1^State Key Laboratory of Environmental Geochemistry, Institute of Geochemistry, Chinese Academy of SciencesGuiyang, China; ^2^Institute of Geochemistry, University of Chinese Academy of SciencesBeijing, China; ^3^The Key Laboratory of Biotechnology for Medicinal Plant of Jiangsu Province, School of Life Science, Jiangsu Normal UniversityXuzhou, China; ^4^Division of Earth and Ecosystem Sciences, Desert Research InstituteLas Vegas, NV, USA; ^5^State Key Laboratory of Microbial Resources, Institute of Microbiology, Chinese Academy of SciencesBeijing, China; ^6^Department of Biotechnology, College of Life Science, Nanjing Normal UniversityNanjing, China

**Keywords:** lithophilous actinobacteria, *Streptomyces luteogriseus* DHS C014, biomineralization, hexagonal prism calcite, doughnut-like vaterite

## Abstract

Microorganisms and their biomineralization processes are widespread in almost every environment on earth. In this work, *Streptomyces luteogriseus* DHS C014, a dominant lithophilous actinobacteria isolated from microbial mats on limestone rocks, was used to investigate its potential biomineralization to allow a better understanding of bacterial contributions to carbonate mineralization in nature. The ammonium carbonate free-drift method was used with mycelium pellets, culture supernatant, and spent culture of the strain. Mineralogical analyses showed that hexagonal prism calcite was only observed in the sub-surfaces of the mycelium pellets, which is a novel morphology mediated by microbes. Hemispheroidal vaterite appeared in the presence of spent culture, mainly because of the effects of soluble microbial products (SMP) during mineralization. When using the culture supernatant, doughnut-like vaterite was favored by actinobacterial mycelia, which has not yet been captured in previous studies. Our analyses suggested that the effects of mycelium pellets as a molecular template almost gained an advantage over SMP both in crystal nucleation and growth, having nothing to do with biological activity. It is thereby convinced that lithophilous actinobacteria, *S. luteogriseus* DHS C014, owing to its advantageous genetic metabolism and filamentous structure, showed good biomineralization abilities, maybe it would have geoactive potential for biogenic carbonate in local microenvironments.

## Introduction

Biomineralization refers to the processes by which living organisms form minerals (Dhami et al., 2013), which happened in the geological record as soon as the prokaryotes appeared about 3.5 Ga ago (Weiner and Dove, 2003). Since then, minerals at the Earth's surface have begun to co-evolve with microbial life (Hazen et al., 2008). As life evolved and diversified, especially with the emergence of the eukaryotes, the diversity of mineral-forming organisms and biominerals rose accordingly. To date, more than 60 biominerals have been identified (Weiner and Dove, 2003). Of these, one of the most significant groups, both in terms of quantity and distribution, is the carbonate minerals. This is not surprising: virtually all living organisms, in one way or another, affect the formation environment of carbonate minerals by either taking up, or giving off, CO_2_, or bicarbonate, and thereby affect the carbonate equilibrium (Lowenstam and Weiner, 1989). Within this group, there are eight calcium carbonate polymorphs, seven of which are crystalline. Of these, three—calcite, aragonite and vaterite—are pure calcium carbonate, and two are monohydrocalcite. Amorphous calcium carbonate, on a *per* mole basis, contains one mole of water (Addadi et al., 2003).

The term “sub-aerial biofilm” (SAB) is used to describe microbial communities that usually develop on mineral surfaces exposed to the atmosphere (Gorbushina, 2007). Attributed to their diversities in physiology and metabolism, microbes are widely considered to play an important role in the formation of carbonate biominerals (Lian et al., 2010; Xiao et al., 2015). Numerous reports exist in the literature of carbonate precipitation mediated by different taxa, including bacteria (Braissant et al., 2003; Lian et al., 2006; Al-Thawadi et al., 2012; Torres et al., 2013; Lee et al., 2014; Srivastava et al., 2015), cyanobacteria (Obst et al., 2009a,b; Couradeau et al., 2012; Kang and Roh, 2013; Uma et al., 2014), fungi (Ahmad et al., 2004; Burford et al., 2006; Hou et al., 2011; Wei et al., 2013), and algae (Hammes and Verstraete, 2002; Holtz et al., 2013; Saghaï et al., 2015). However, the precise principle underpinning biomineralization, as mediated by these microorganisms, remained largely elusive (Dupraz et al., 2009; Couradeau et al., 2012; Ionescu et al., 2014). As a result, compared to carbonate mineral formation in large animals, the extent of biological biomineralization induced by microbes remains a subject of investigation. The roles of living microorganisms generally consist of three different, yet related, routes. Ordered organic molecules on the cell surfaces, such as polysaccharide or lipopolysaccharide, may serve as nucleation sites and help to decrease the activation energy required for initiation of crystal growth. Many organics have negatively charged residues and absorb divalent cations including Ca^2+^ (Schultze-Lam et al., 1996; Rivadeneyra et al., 1998; Kenward et al., 2013), increasing their local concentration. Second, rapid heterotrophic activity releases CO_2_ as a by-product, raising local ${\text{CO}}_{3}^{2-}$ concentrations (Lian et al., 2006). Third and last, the uptake of CO_2_ and bicarbonate by photosynthetic organisms can increase the local pH (Dupraz et al., 2009). As a result of such activities, the saturation index of carbonate can be significantly different from that of the bulk environment, leading to local precipitation of calcium carbonate on the growing organisms.

In 2012, our team had already studied the phylogenetic diversities of endolithic bacterial communities on limestone rocks using a restriction fragment length polymorphism (RFLP) method, which demonstrated that large percentages of bacterial clones were related to the *Actinobacteria, Alphaproteobacteria*, and *Cyanobacteria* (Tang et al., 2012). *Actinobacteria* is a morphologically diverse phylum of Gram-positive bacteria (Cockell et al., 2013), and plays a crucial role in matter cycling as a decomposer. It is thought to be one of the primary phyla to colonize terrestrial surfaces for its evolution some 2.7 Ga or so (Battistuzzi et al., 2004; Battistuzzi and Hedges, 2009; Gorbushina and Broughton, 2009). Yet little is known about the role of *Actinobacteria* in carbonate mineral formation (Rautaray et al., 2004; Cockell et al., 2013). Here, 25 pure cultures of actinobacteria were isolated from limestone rocks using selective isolation media according to protocols described in the International *Streptomyces* Project (Shirling and Gottlieb, 1966). Of these, some rare actinobacteria are novel species (Cao et al., 2015), while strain DHS C014 frequently appeared on all media as a dominant actinobacterial species and was therefore used to evaluate its carbonate biomineralization potential. In this study, it showed dramatic differences in morphology and polymorphism of biomineral precipitation.

## Materials and methods

### Sample site and actinobacteria

**Figure 1:**
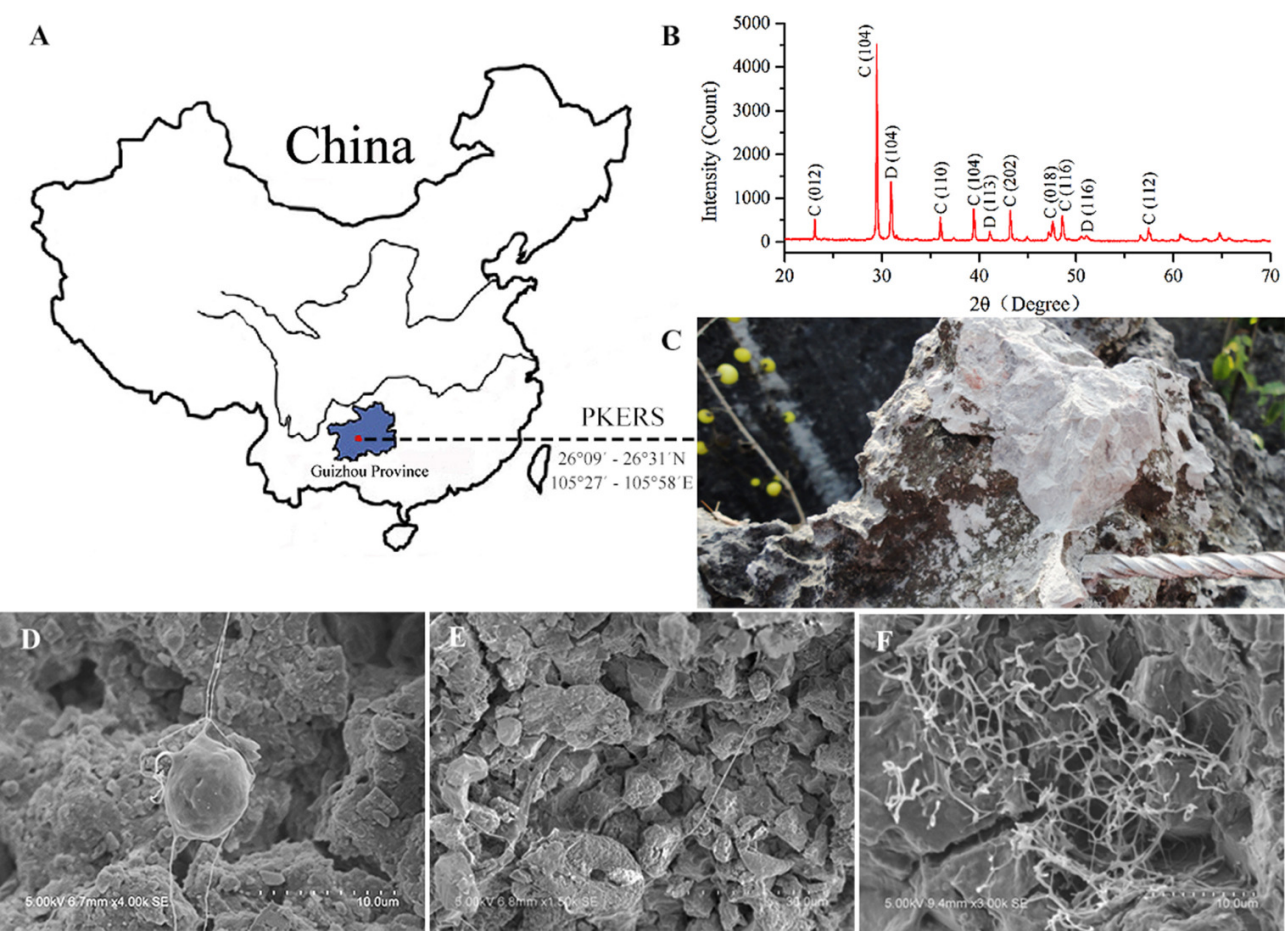
**Map of Puding Karst Ecosystem Research Station (PKERS) and geochemical analyses of the mineral samples by XRD and SEM**. Panel **(A)** Showing the sampling site; **(B)** showing X-ray powder diffraction patterns of the mineral samples [Numbers in the parentheses indicate the Miller indices, whereas C and D denote calcite and dolomite, respectively]; **(C)** showing the microbial mats on limestone rocks; **(D–F)** showing filamentous microorganisms on limestone rocks.

Limestone samples used for microbial isolation were collected at the Puding Karst Ecosystem Research Station (PKERS) of the Chinese Academy of Sciences in Guizhou Province, China (26°09′–26°31′N, 105°27′–105°58′E; Figure 1A). X-ray powder diffraction data (XRD Bruker D8-ADVANCE) showed that calcite was the dominant mineral phase of these limestone samples (Figure 1B). As shown in Figure 1C, limestone rocks were almost completely covered with microbial mats. In detail, many filamentous microorganisms living on the limestone were observed using scanning electron microscopy (SEM, Hitachi S-3400N; Figures 1D–F). X-ray fluorescence spectroscopy (XRF Bruker S8-TIGER equipped with 4 kW, Rh anode X-ray tube) showed that CaO, MgO, SiO_2_, Fe_2_O_3_, Al_2_O_3_, and CO_2_ accounted for 51.40, 3.99, 1.02, 0.23, 0.22, and 42.03% of the limestone by mass, respectively (data were expressed as oxides).

The morphological properties of strain DHS C014 were examined by SEM using cultures grown on ISP 2 medium at 28°C for 21 days. Extraction of genomic DNA and 16S rRNA gene amplification were carried out according to the procedures described by Qin et al. (2009). The almost complete 16S rRNA gene sequence of the strain was subjected to BLAST sequence similarity search from the GenBank and EzTaxon-e databases (Kim et al., 2012). Phylogenetic trees between the isolated, and closely-related, strains were inferred using a neighbor-joining tree algorithm (MEGA software, Version 5.0) with bootstrap values based on 1000 repeats (Felsenstein, 1985; Saitou and Nei, 1987).

**Figure 2:**
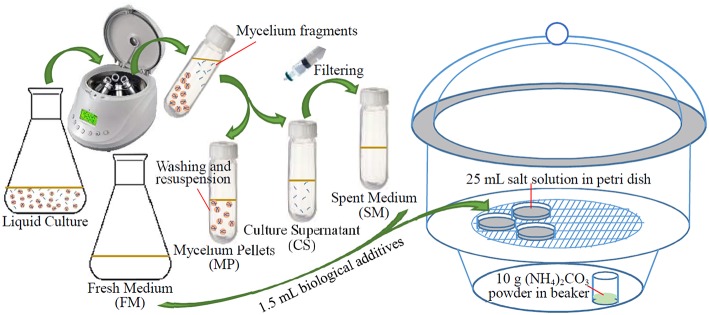
**Schematic diagram of the experimental procedure**.

The strain was inoculated in 500 mL Erlenmeyer flasks containing 100 mL malt extract-glucose-yeast extract-peptone (MGYP) medium that consisted of: malt extract 0.3%, glucose 1%, yeast extract 0.3%, and peptone 0.5% (Rautaray et al., 2004). After adjusting the pH of the medium to 7.2 (6.9 after autoclave treatment), the cultures were incubated under continuous shaking on a rotary shaker (180 rpm) at 28°C for about 120 h, until microbial cells reached their late exponential phase. Mycelium pellets were harvested by centrifugation at 2000 rpm for 15 min at 4°C. Biological additives used in this study included: (i) Fresh medium (FM, as controls); (ii) Mycelium pellets (MP, mycelium pellets harvested by centrifugation were washed and re-suspended with sterile distilled water); (iii) Culture supernatant (CS, without mycelium pellets but including small mycelium fragments and other residues); (iv) Spent medium (SM, the culture supernatant was further filtered with a 0.22 μm sterilized membrane to eliminate mycelium fragments and other residues). The general procedure is shown in Figure 2.

### Biomineralization experiments

Biomineralization experiments were conducted with the ammonia free-drift method described by Lian et al. (2006). The experiments were performed in Petri dishes which were enclosed in a large desiccator (Figure 2). The Petri dishes contained 25 mL salt solution prepared by mixing equal volumes of reagent grade NaHCO_3_ (2 mM) and Ca(NO_3_)_2_·4H_2_O (2 mM) in deionized distilled water: the pH of salt solution was adjusted to ~3 using HCl (approx. 2 M) to ensure that no deposit appeared. About 10 g of (NH_4_)_2_CO_3_ powder was placed in bottom of the desiccator. Chemical reactions are as follows:

(1) $$\begin{array}{r} {\left({\text{NH}}_{4}\right)}_{2}{\text{CO}}_{3}\to 2{\text{NH}}_{3}↑+\;{\text{CO}}_{2}↑+\;{\text{H}}_{2}\text{O} \end{array}$$

(2) $$\begin{array}{r} {\text{NH}}_{3}\;+\;{\text{H}}_{2}\text{O}↔{\text{NH}}_{4}^{+}+\text{O}{\text{H}}^{-} \end{array}$$

(3) $$\begin{array}{r} {\text{Ca}}^{2+}+\text{HC}{\text{O}}_{3}^{-}↔{\text{CaCO}}_{3}↓+\;{\text{H}}^{+} \end{array}$$

The NH_3_ gas from the chemical decomposition of (NH_4_)_2_CO_3_, rapidly dissolved into the mineralization solution with a resultant pH increase. These reactions create carbonate alkalinity, which is one of the two factors affecting the Saturation Index (SI) defined as:

$$\begin{array}{l} \text{SI}=\log\left(\text{IAP}∕K_{\text{SP}}\right) \end{array}$$

Where IAP denotes the ion activity product, that is {Ca^2+^} × {${\text{CO}}_{3}^{2-}$}, and *K*_SP_, the solubility product of the corresponding mineral (Dupraz et al., 2009).

Petri dishes were inoculated with 1.5 mL of biological additives. Each treatment was run in triplicate, at 28°C for 7 days. When biomineralization was completed, minerals and glass cover-slips in the Petri dishes were collected and washed twice with double-distilled water. These air-dried samples were prepared for morphological, and polymorphic, analyses.

### Polymorphism analyses

XRD patterns were registered using a Bruker D8 Advance diffractometer with a Cu target Kα radiation source (accelerating voltage of 40 kV) at a scan speed of 0.1 s/step and a step scan of 0.02° (10 ≤ 2θ ≤ 90°). Fourier transform infrared scanning (FTIR Thermo iS10) is another useful tool for identification of CaCO_3_ polymorphs. FTIR spectra were collected at room temperature with KBr discs in the 400–2000 cm^−1^ region.

### Biomineral morphology

After gold coating to a thickness of ~15 nm (Hitachi E-1010), the glass cover-slips were examined by SEM, using a secondary electron detector with a 5–15 kV accelerating voltage. Compositional analyses were performed using energy dispersive spectroscopy (EDS, Horiba EMAX 7021-H) at a 10 mm working distance and a 15 kV accelerating voltage.

## Results and discussion

### Identification of strain DHS C014

**Figure 3:**
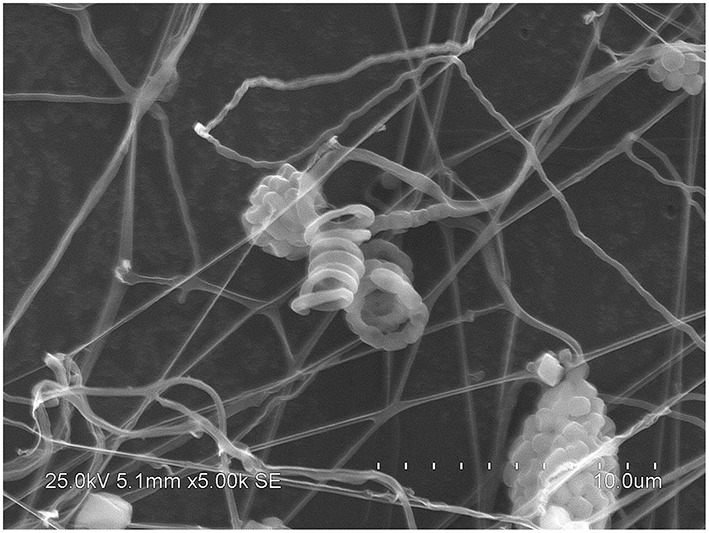
**Scanning electron micrograph of strain DHS C014**. It shows aerial mycelia fragmenting into spiral spore chains after growth on ISP 2 agar at 28°C for 21 days. Bar, 10 μm.

**Figure 4:**
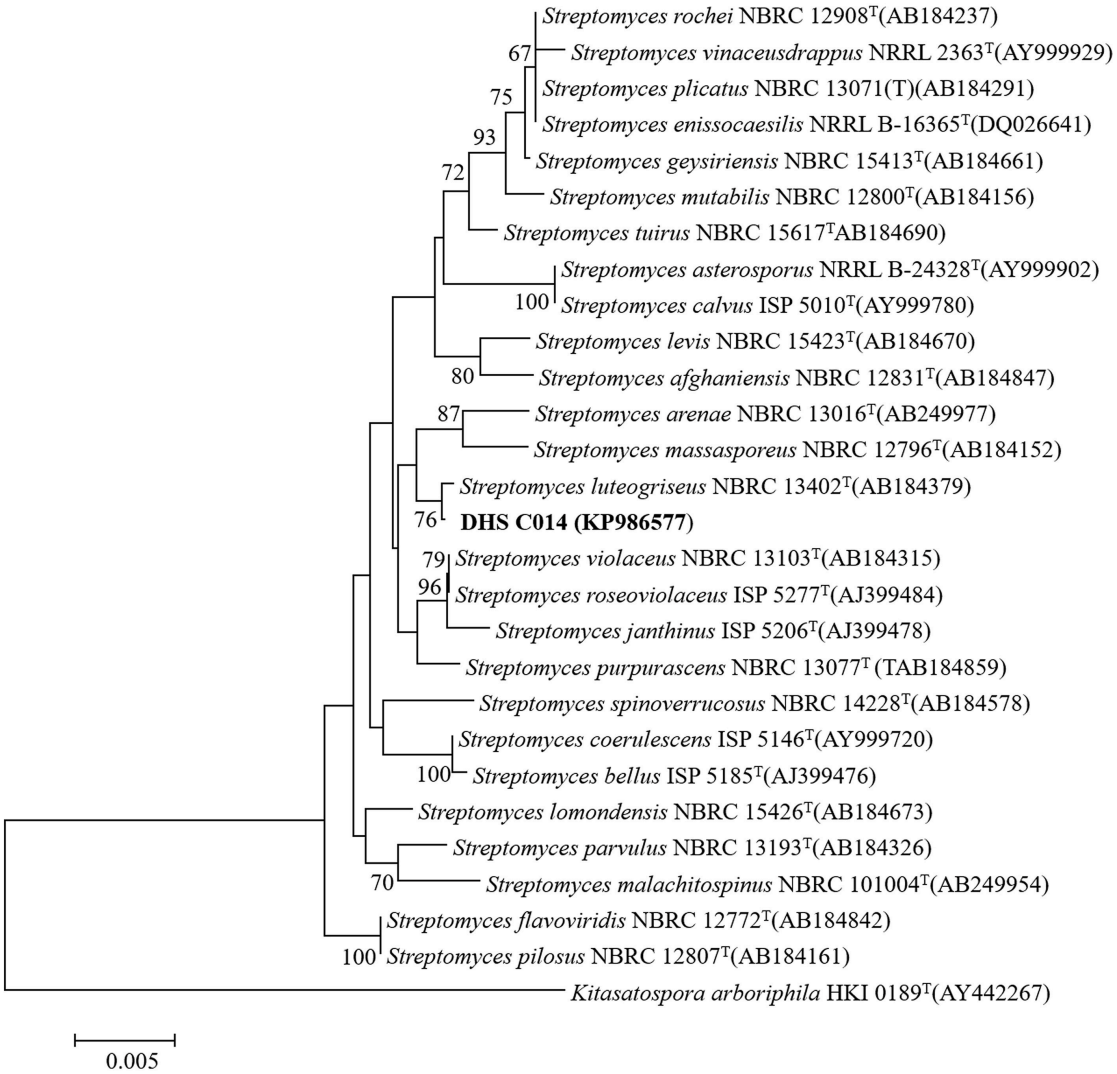
**Neighbor-joining tree based on 16S rRNA gene sequences**. It shows the relationships between strain DHS C014 and partial species of the genus *Streptomyces*. The sequence of *Kitasatospora arboriphila* HKI0189T (AY442267) was used as out-group. Numbers at branch nodes are bootstrap values (1000 re-samplings). Bar, 0.005 sequence variation.

After incubation on ISP 2 agar at 28°C for 21 days, aerial mycelia usually crimped into spiral spore chains, and some of them began to fragment into short rod-shape spores with smooth surfaces (Figure 3). The almost complete 16S rRNA gene (1475 bp) of the strain was sequenced and deposited in GenBank with accession number KP986577. The strain shared its highest levels of 16S rRNA gene sequence similarity with the closest type strain *Streptomyces luteogriseus* NBRC 13402^T^ (99.9%), and for other species of the genus the similarities were below 99.5%. The phylogenetic tree, based on the neighbor-joining algorithm (Figure 4), showed that strain DHS C014 formed a distinct sub-branch with the closest types strain, *S. luteogriseus* NBRC 13402^T^, supported by a bootstrap value of 76%. Based on the morphological and genotypical properties, the strain was identified as *S. luteogriseus* DHS C014.

### Polymorphic analyses

**Figure 5:**
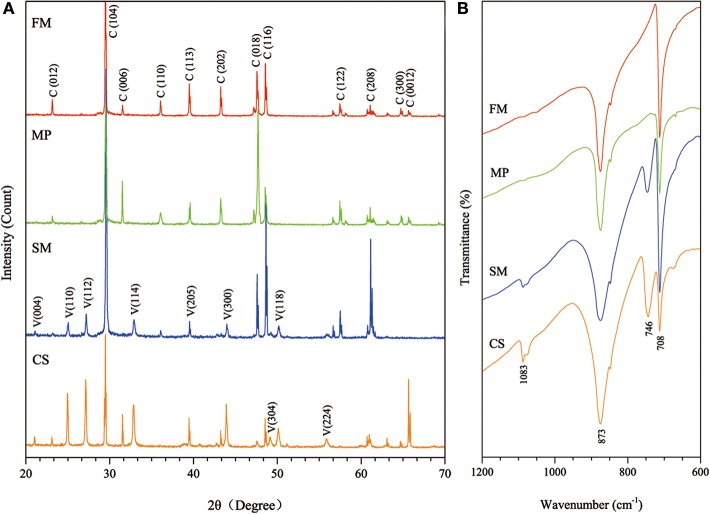
**Mineralogical analyses of biominerals collected from the four experimental treatments**. **(A)** Showing XRD patterns of the biominerals (numbers in parentheses indicate the Miller indices, whereas C and V denote calcite and vaterite, respectively); **(B)** showing FTIR spectra of the biominerals (FM: fresh medium, MP: mycelium pellets, SM: spent medium, CS: culture supernatant).

*S. luteogriseus* DHS C014 showed special CaCO_3_ biomineralization *in vitro*. After incubation for 7 days, the pH of all treatments increased from 3.2–3.5 to 8.7–9.2. Calcite contents were determined in all experiments, which showed characteristic peaks in their XRD profiles, including Miller indices (012), (104), (006), (110), (113), (202), (018), and (116; Figure 5A). This indicated that the chemical cause of calcite generation was the increase in pH during mineralization.

Vaterite was present in SM and CS treatments with characteristic XRD peaks, e.g., (110), (112), (114), (205), (300), (304), (118), and (224). Consistent with XRD analyses, FTIR spectra showed that the absorption bands of calcite at 708 and 873 cm^−1^ (υ_4_ and υ_2_, respectively), whereas 746 and 1083 cm^−1^ (υ_4_ and υ_2_, respectively) were characteristic of vaterite (Figure 5B). Negatively charged organic molecules produced by microorganisms were probably responsible for vaterite precipitation. As described earlier, spheroidal vaterite formed in the presence of soil bacterium *Myxococcus xanthus* (Rodriguez-Navarro et al., 2007). In contrast, Tourney and Ngwenya concluded that EPS extracted from *Bacillus licheniformis* could inhibit vaterite formation during biomineralization, and only calcite appeared in the end (Tourney and Ngwenya, 2009).

In these experiments, vaterite present in SM and CS treatments was stable, and was not transformed to calcite after at least 7 days. Electrostatic attraction between Ca^2+^ and biomacromolecules (e.g., silk fibroin) probably contributes to the stability of vaterite (Liu et al., 2015). So, it is safe to draw the conclusion that this was also the case with strain DHS C014. The presence of soluble microbial products (SMP) acts a template and creates a local environment, which may favor the attraction of Ca^2+^, and gradually reaching carbonate saturation (Tourney and Ngwenya, 2014). Yet much remains to be revealed about the mechanisms underpinning the ways in which acidic organic molecules (such as polysaccharides, proteins, or amino acids) affect biomineral composition, microstructure, shape, and size (Kröger, 2015).

### Mineral morphology

**Figure 6:**
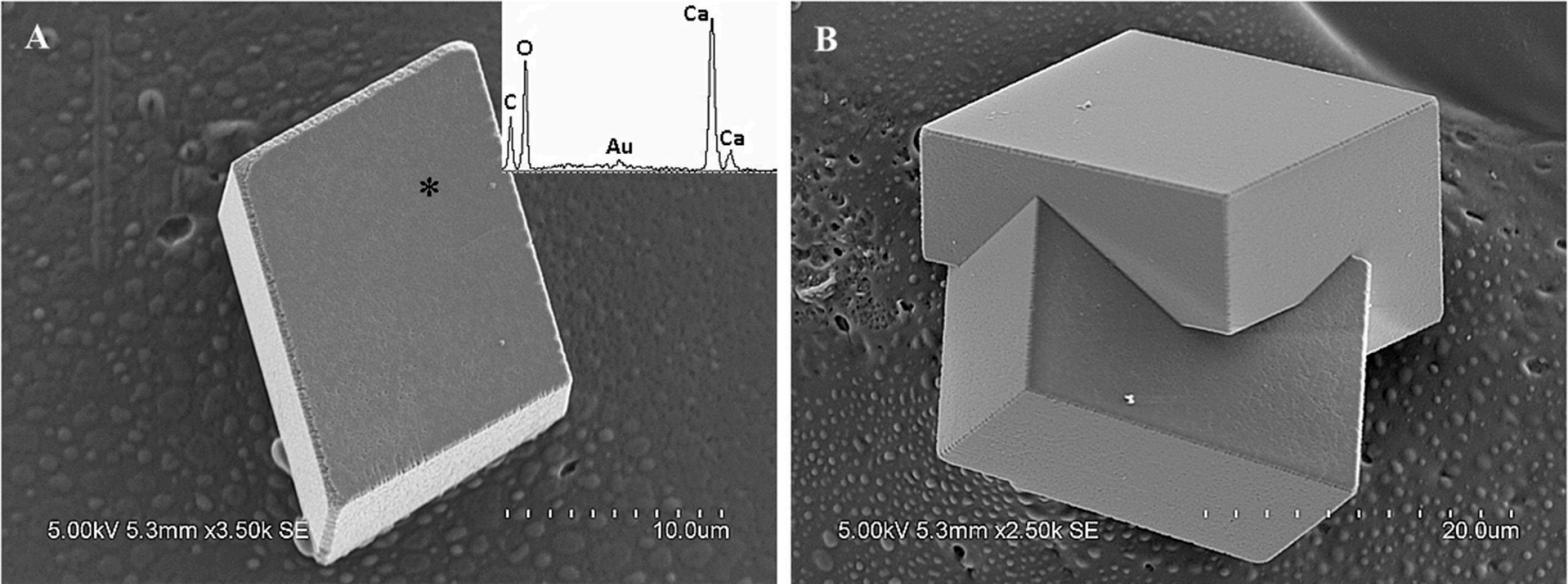
**Morphologies of biominerals collected from FM treatments**. Panel **(A)** Showing rhombohedral calcite and EDS spectrum obtained from asterisk site on (104) face (the Au peak was the result of the ion sputtering used before SEM examination); **(B)** showing polyhedral calcite twins.

Calcite crystals in FM treatments displayed a characteristic rhombohedral morphology (Figure 6A). Sometimes, few contact twins also appeared (Figure 6B). The crystals, ranging from sizes of 10–35 μm, have well-defined faces and edges with perfect cleavages on their (104) faces. The asterisked site on the (104) face shown in Figure 6A denoted the sampling point for EDS analysis. The EDS profile showed that Ca, C, and O were the major elements, and Au peak was due to the ion sputtering used before SEM examination. In the presence of biological additives, however, rhombohedral calcite was occasionally observed, mainly because that chemical cause, when used in the free-drift method, usually interfered with the biological contribution to mineralization.

**Figure 7:**
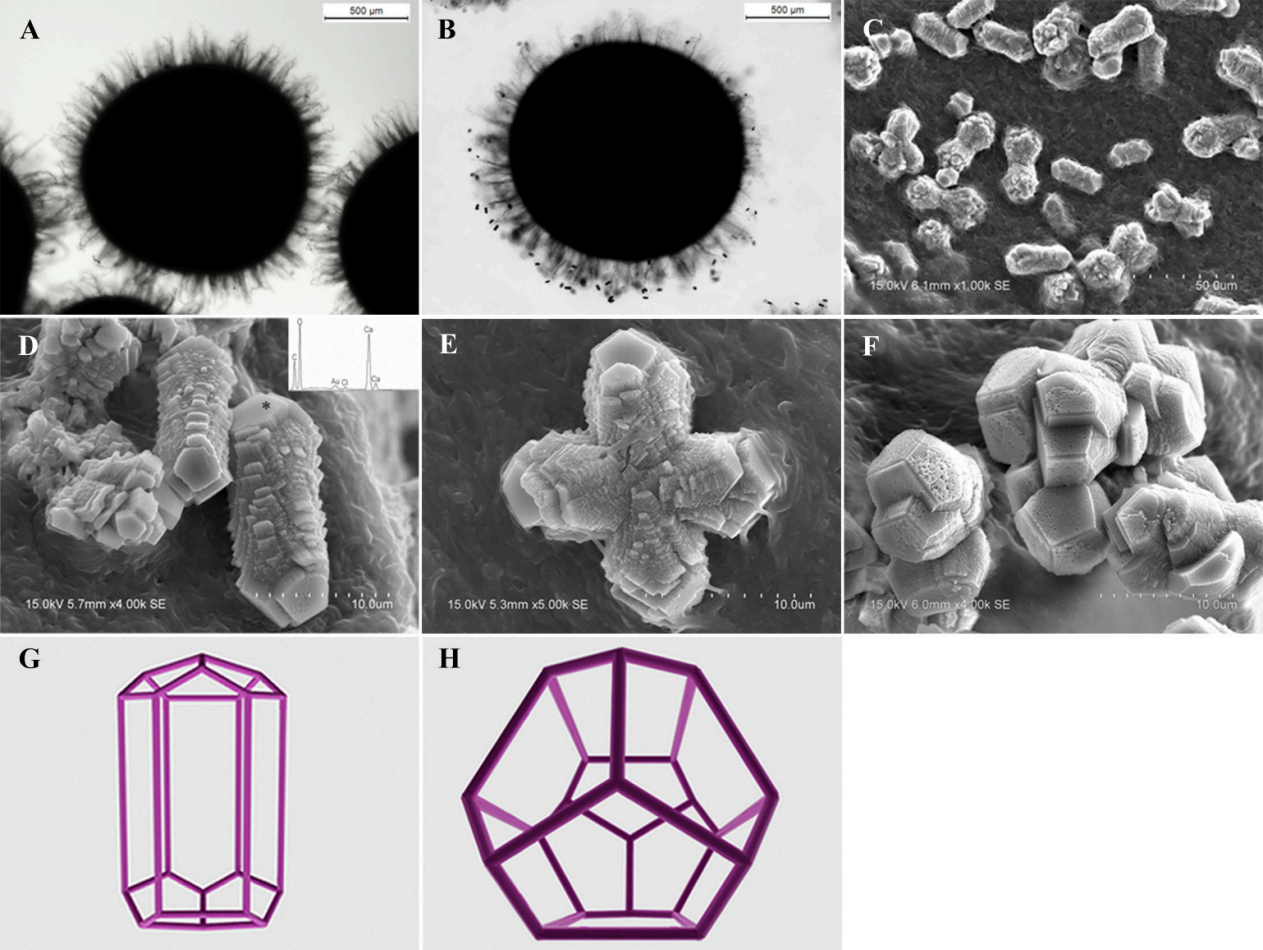
**Morphologies of biominerals in MP treatments**. **(A,B)** Optical micrographs of mycelia pellets before/after mineralization, respectively; **(C)** showing crystals with homogeneous morphology in full view; **(D)** showing the details of most crystals with hexagonal prism morphology and EDS spectrum obtained from the asterisked site on the (018) face (the Au peak was the result of ion sputtering used before SEM examination); **(E,F)** showing calcite twins and polycrystals; **(G,H)** showing a three-dimensional diagram simulating the hexagonal prism calcite with front and top views, respectively.

In MP treatments, calcite was prone to nucleate in the sub-surfaces of mycelium pellets. At the end of mineralization, these mycelium pellets (Figure 7A) observed using optical microscopy (Leica DM500B) were covered with lots of rod-shaped crystals (Figure 7B). These near-developed calcite crystals showed a hexagonal prism shape as seen upon further observation by SEM: these were significantly different from rhombohedral crystals in FM treatments. It is a novel morphology of calcite mediated by microbes, somewhat similar to the sodium salt of poly L-isocyanoalanyl-D-alanine as a crystallization template for CaCO_3_ (Donners et al., 2002). Most of crystals were elongated along the crystallographic c-axis with three end faces (018) expressed on each side of the crystal (Figures 7C,D). The well-defined (018) faces mostly showed sound edges, but the (100) faces and their edges were not completely developed (Figure 7D). Ca, C, and O were identified as the major elements in the hexagonal prism crystals (Figure 7D) by EDS, which was the same as that of rhombohedral calcite in FM. Sometimes, a few crystals could develop into contact twins (Figure 7E) and polycrystals (Figure 7F), presumably due to induction and steric hindrance of complex template structures. The hexagonal prism morphology of calcite was simulated using 3D Studio Max software (Autodesk 2014), giving a front view (Figure 7G), and top view (Figure 7H). The same crystals were also found in trial treatments for biomineralization in the presence of mycelium pellets which were treated by autoclaving at 121°C for 30 min. This demonstrated that the effect of the molecular template associated with the microbial cell-walls played a conspicuous role in crystal nucleation and growth, having nothing to do with biological activity. In previous studies, different microbial cells could induce rhombohedral calcite (Lian et al., 2006), vaterite covering cells (Rodriguez-Navarro et al., 2007), spherical vaterite (Tourney and Ngwenya, 2009), peanut-like vaterite (Chen et al., 2008), respectively (Table 1).

**Table 1:** **Overview of Ca-carbonate precipitates mediated by different bacteria (part of the bacteria investigated in other researches)**.

**Crystal precipitation mediated by microbial cells**	**Crystal precipitation mediated by SMP**	**Crystal precipitation mediated by combination of microbial cells and SMP**	**Bacteria and references**
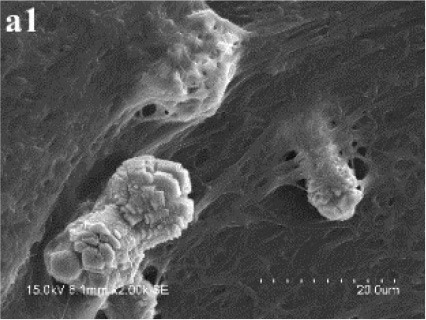 Calcite	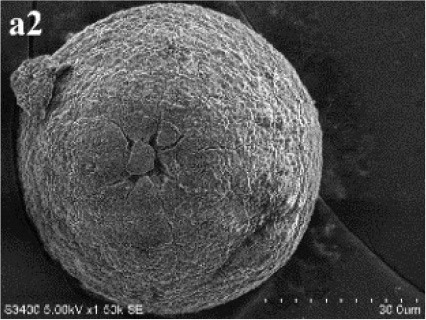 Vaterite	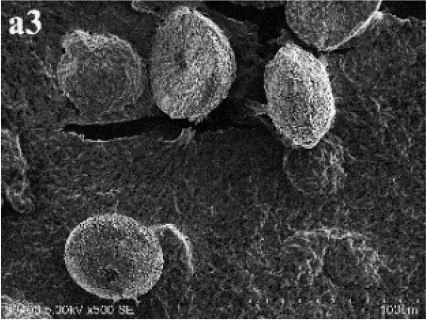 Vaterite	*S. luteogriseus* DHS C014, this study
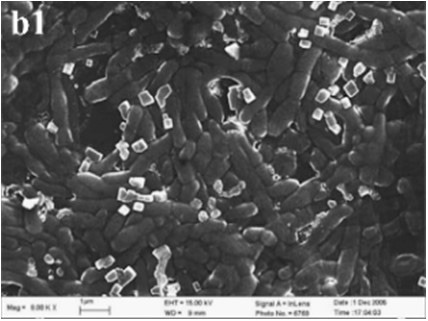 Calcite	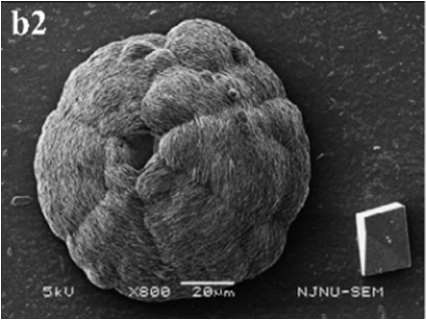 Vaterite	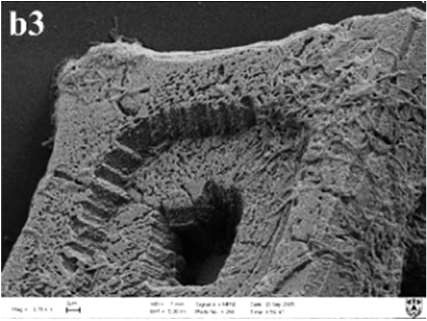 Calcite	*Bacillus megaterium*, (Lian et al., 2006)
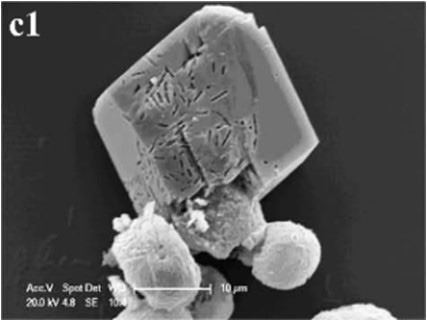 Vaterite and calcite (48 h)	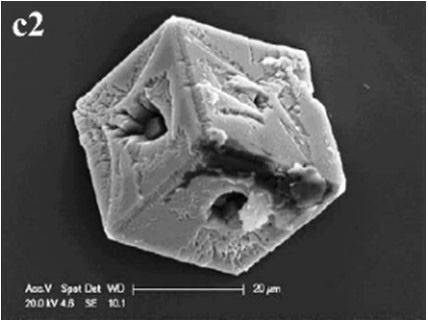 Calcite (48 h)	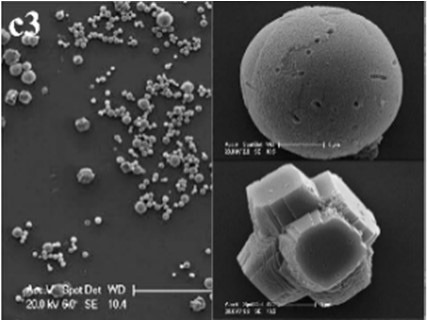 Vaterite and calcite (48 h)	*Bacillus licheniformis*, (Tourney and Ngwenya, 2009)
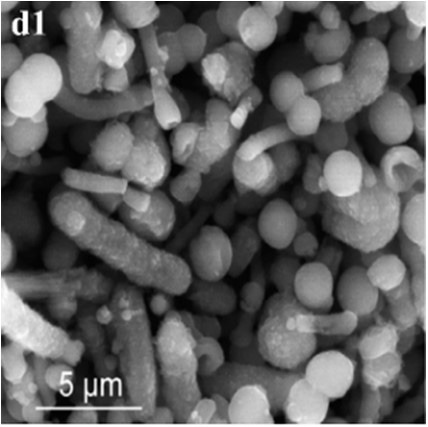 Calcified rod-shaped bacterial cells with vaterite	No	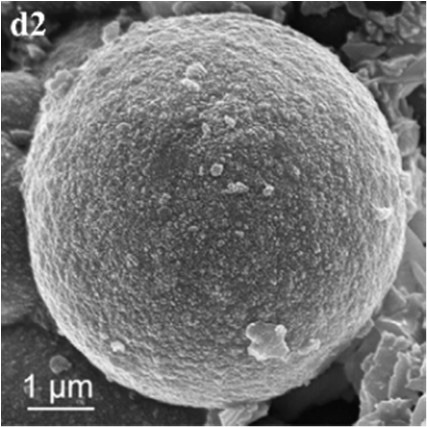 Vaterite	*Myxococcus xanthus*, (Rodriguez-Navarro et al., 2007)
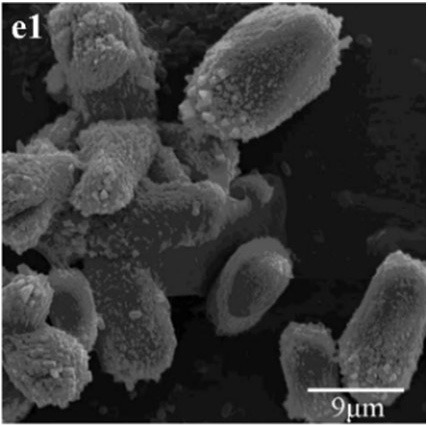 Vaterite	No	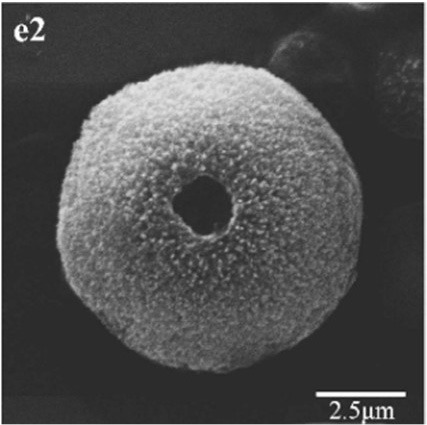 Vaterite	*Proteus mirabilis*, (Chen et al., 2008)

*Differences in morphology and polymorphism were related to microbial cells, SMP, and combinations of these two factors, respectively. See text for details*.

**Figure 8:**
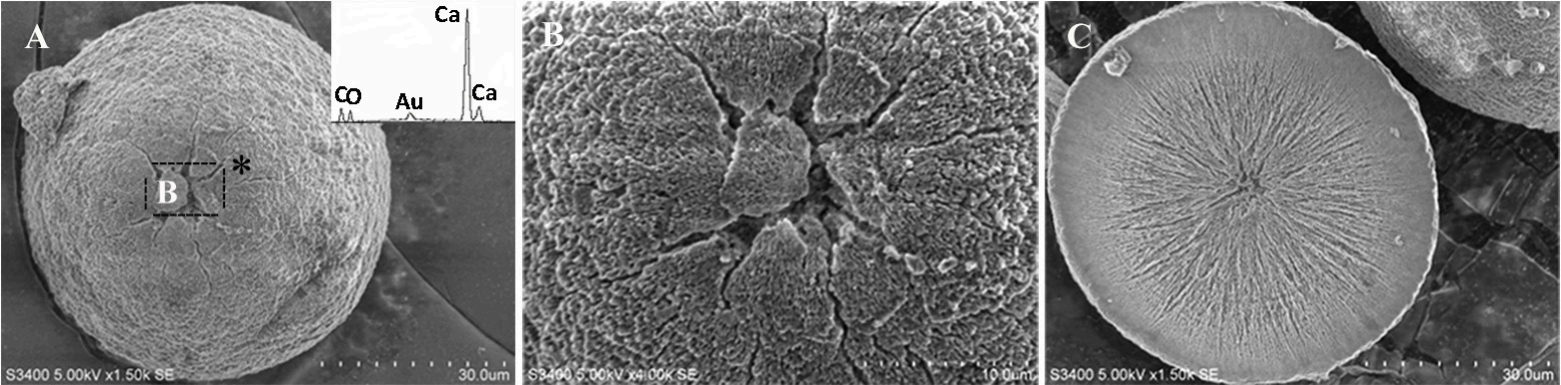
**Morphologies of biominerals in SM treatments**. Panel **(A)** Showing vaterite with spherical cone morphology and EDS spectrum obtained from the asterisked site (the Au peak was the result of ion sputtering used before SEM examination); **(B)** showing crystal details of the boxed area on Panel **(A)**; **(C)** showing bottom details of the biominerals.

In SM treatments, vaterite crystals were characteristically hemispheroidal in the presence of spent medium (Figure 8A). Several radial flaws appeared on the top of the hemispheroidal crystal (Figure 8B). EDS data identified Ca, C, and O as major elements in the spherical cone crystals (Figure 8B), in agreement with XRD and FTIR data. On the other hand, the bottom of the crystals developed into a fibro-radial structure with slight central invagination (Figure 8C). Hemispheroidal vaterite crystals in SM treatments were different from those seen in previous studies in mesocrystals and their reorganization into larger crystals. In the presence of a super-solution of *Bacillus megaterium*, spherulitic vaterite with a hollow core seemed to be composed of six identical cloves (Table 1b2; Lian et al., 2006). Vaterite spherulites with smoother surfaces (Table 1d2) were induced during the incubation of *M. xanthus* at 28°C with constant shaking (Rodriguez-Navarro et al., 2007). Whereas, vaterite spheres with a hole on their surfaces (Table 1e2) were mediated by *Proteus mirabilis* growing in a reaction solution (0.1 mol L^−1^ CaCl_2_ and 0.2 mol L^−1^ urea) at 27°C for 5 days (Chen et al., 2008). During the reorganization process, small aggregated 20–30 nm nanoparticles resulted in rough mesocrystal surfaces to develop. The crystal surfaces perhaps became covered with holes due to specific protein binding, and subsequent inhibition of crystal growth (Mann et al., 2007; Decho, 2010). Similarly, Rodriguez-Navarro concluded that surfaces of these biominerals were very rough in the presence of aggregates of nanometer-sized building blocks (Rodriguez-Navarro et al., 2007, 2012). These results pertinent to the morphology and polymorphism of biominerals suggested that mineralization mediated by microbes, to some extent, was strain-specific and associated with various biomacromolecular templates.

**Figure 9:**
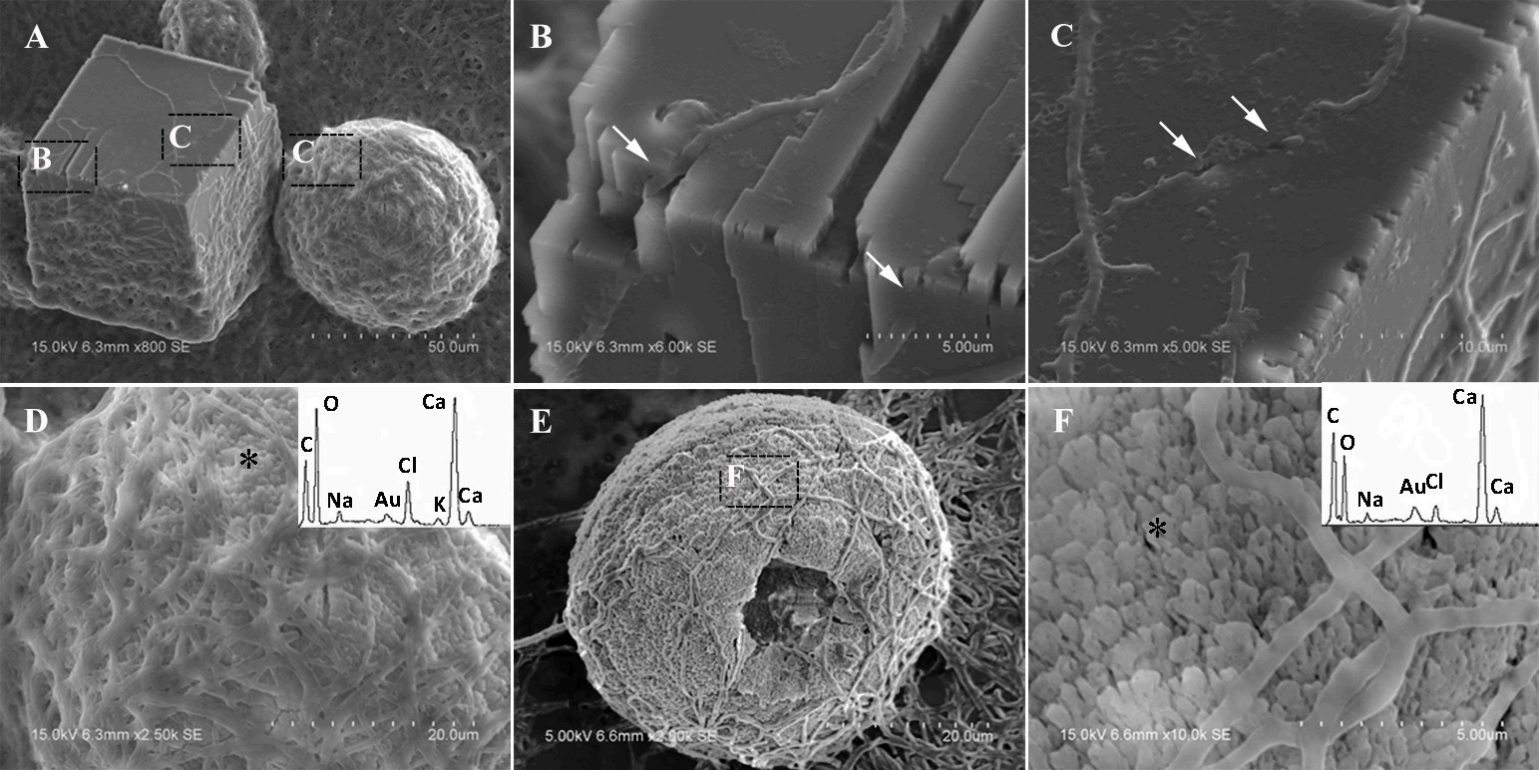
**Morphologies of biominerals in CS treatments**. Panel **(A)** Showing calcite with rhombohedral morphology and vaterite with hemispheroid morphology; **(B,C)** showing crystal details of calcite areas enclosed by dashed boxes on Panel **(A)**; **(D)** showing crystal details of vaterite area enclosed by a dashed box on Panel **(A)** and EDS spectrum obtained from the asterisked site (the Au peak was the result of ion sputtering used before SEM examination); **(E)** showing doughnut-like vaterite; **(F)** showing details of the boxed area on Panel **(E)** and EDS spectrum obtained from the asterisked site.

In CS treatments, an interesting aspect was that a rhombohedral calcite appeared in close contact with a hemispheroidal vaterite (Figure 9A). The following magnified images show that mycelia spread over the crystal surfaces, and were occasionally buried inside the crystal (arrows in Figures 9B,C). In addition, the crystal displayed its angles and edges of its (104) face with perfect cleavages. The vaterite crystal was fully covered by a network of mycelium, and it was hard to observe any details (Figure 9D). Furthermore, a good many doughnut-like crystals (Figure 9E) frequently appeared in this case. EDS data showed that Ca, C, and O were the major elements in these two shapes of crystals (Figures 9D,F). Na, K, and Cl, were introduced from the microbes. During mineralization process, these crystals were twinned with mycelia and braced firmly onto the mycelial mats. Thanks to this mycelial assistance, crystals here could easily develop from hemispheroid into spheroid form. However, the mycelia growing on these crystals can also hinder further crystal growth, and finally contributed to the doughnut-like crystals with their axial invagination. Unicellular bacteria described earlier could not contribute to the precipitation of doughnut-like crystals. In this case, small mycelia fragments could survive and grow slowly: this had less effects on the polymorphism than the SMP in the solution. On the contrary, another experiment was conducted that biomineralization occurred in the presence of washed mycelium pellets and spent culture. The results were consistent with MP treatments, indicating that mycelium pellets as a molecular template gained an advantage over SMP both in crystal nucleation and growth.

## Conclusions

In this study, *S. luteogriseus* DHS C014, a dominant lithophilous actinobacteria isolated from microbial mats on limestone rocks, was used to investigate its potential biomineralization *in vitro*, especially to evaluate the contribution of mycelia, SMP, and their combined action to mineral morphologies and polymorphs.

The analysis suggested that mycelium pellets of *S*. *luteogriseus* DHS C014, used as templates, could induce precipitation of hexagonal-prism calcite, which is a novel morphology mediated by microbes. The same crystals were also mediated by autoclaved mycelium pellets, indicating that it had nothing to do with biological activity, but was an effect arising from the templating. Whereas, vaterite appeared in the presence of spent culture or culture supernatant, mainly because of the action of SMP during mineralization. Hemispheroidal vaterite crystals present in SM treatments were different from those found in previous studies in mesocrystals, and in their reorganization into larger crystals. Especially in CS treatments, doughnut-like vaterite, favored by actinobacterial mycelia, has not yet been recorded in previous studies. When in the presence of mycelium pellets and spent culture, mycelium pellets as a molecular template, almost gained an advantage over SMP both in crystal nucleation and growth.

Based on the results in this study, it may be concluded that *S*. *luteogriseus* DHS C014, owing to its advantages both in genetic metabolism and its filamentous structure, showed good biomineralization abilities, and maybe had geoactive potential for biogenic carbonate in local microenvironments.

## Author contributions

BL and FT designed this study. CC performed the laboratory work. BL, CC, HS, JJ, and YH analyzed the data. BL, CC, and HS wrote this manuscript. All authors have read and approved the final manuscript.

## Funding

This study was jointly supported by grants from the National Science Foundation of China (Grant No. 41373078), the National Key Basic Research Program of China (Grant No. 2013CB956702), and Natural Science Foundation of Jiangsu Normal University (13XLA02).

### Conflict of interest statement

The authors declare that the research was conducted in the absence of any commercial or financial relationships that could be construed as a potential conflict of interest.

## References

[B1] Addadi L. Raz S. Weiner S. (2003). Taking advantage of disorder: amorphous calcium carbonate and its roles in biomineralization. Adv. Mater. Weinheim. 15, 959–970. 10.1002/adma.200300381

[B2] Ahmad A. Rautaray D. Sastry M. (2004). Biogenic calcium carbonate: calcite crystals of variable morphology by the reaction of aqueous Ca^2+^ ions with fungi. Adv. Funct. Mater. 14, 1075–1080. 10.1002/adfm.200400005

[B3] Al-Thawadi S. Cord-Ruwisch R. Bououdina M. (2012). Consolidation of sand particles by nanoparticles of calcite after concentrating ureolytic bacteria *in situ*. Int. J. Green Nanotechnol. 4, 28–36. 10.1080/19430892.2012.654741

[B4] Battistuzzi F. U. Hedges S. B. (2009). A major clade of prokaryotes with ancient adaptations to life on land. Mol. Biol. Evol. 26, 335–343. 10.1093/molbev/msn247 18988685

[B5] Battistuzzi F. U. Feijao A. Hedges S. B. (2004). A genomic timescale of prokaryote evolution: insights into the origin of methanogenesis, phototrophy, and the colonization of land. BMC Evol. Biol. 4:44. 10.1186/1471-2148-4-44 15535883 PMC533871

[B6] Braissant O. Cailleau G. Dupraz C. Verrecchia A. P. (2003). Bacterially induced mineralization of calcium carbonate in terrestrial environments: the role of exopolysaccharides and amino acids. J. Sediment. Res. 73, 485–490. 10.1306/111302730485

[B7] Burford E. P. Hillier S. Gadd G. M. (2006). Biomineralization of fungal hyphae with calcite (CaCO_3_) and calcium oxalate mono-and dihydrate in carboniferous limestone microcosms. Geomicrobiol. J. 23, 599–611. 10.1080/01490450600964375

[B8] Cao C. L. Zhou X. Q. Qin S. Tao F. X. Jiang J. H. Lian B. (2015). Lentzea guizhouensis sp. nov., a novel lithophilous actinobacterium isolated from limestone from the Karst area, Guizhou, China. Antonie Van Leeuwenhoek 108, 1365–1372. 10.1007/s10482-015-0589-x 26377575

[B9] Chen L. Shen Y. Xie A. Huang B. Jia R. Guo R. (2008). Bacteria-mediated synthesis of metal carbonate minerals with unusual morphologies and structures. Crystal Growth Des. 9, 743–754. 10.1021/cg800224s

[B10] Cockell C. S. Kelly L. C. Marteinsson V. (2013). *Actinobacteria*—an ancient phylum active in volcanic rock weathering. Geomicrobiol. J. 30, 706–720. 10.1080/01490451.2012.758196

[B11] Couradeau E. Benzerara K. Gérard E. Moreira D. Bernard S. Brown G. E.. (2012). An early-branching microbialite cyanobacterium forms intracellular carbonates. Science 336, 459–462. 10.1126/science.1216171 22539718

[B12] Decho A. W. (2010). Overview of biopolymer-induced mineralization: what goes on in biofilms? Ecol. Eng. 36, 137–144. 10.1016/j.ecoleng.2009.01.003

[B13] Dhami N. K. Reddy M. S. Mukherjee A. (2013). Biomineralization of calcium carbonates and their engineered applications: a review. Front. Microbiol. 4:314. 10.3389/fmicb.2013.00314 24194735 PMC3810791

[B14] Donners J. J. J. M. Nolte R. J. M. Sommerdijk N. A. J. M. (2002). A shape-persistent polymeric crystallization template for CaCO_3_. J. Am. Chem. Soc. 124, 9700–9701. 10.1021/ja0267573 12175216

[B15] Dupraz C. Reid R. P. Braissant O. Decho A. W. Norman R. S. Visscher P. T. (2009). Processes of carbonate precipitation in modern microbial mats. Earth Sci. Rev. 96, 141–162. 10.1016/j.earscirev.2008.10.005

[B16] Felsenstein J. (1985). Confidence limits on phylogenies: an approach using the bootstrap. Evolution 39, 783–791. 10.2307/2408678 28561359

[B17] Gorbushina A. A. (2007). Life on the rocks. Environ. Microbiol. 9, 1613–1631. 10.1111/j.1462-2920.2007.01301.x 17564597

[B18] Gorbushina A. A. Broughton W. J. (2009). Microbiology of the atmosphere-rock interface: how biological interactions and physical stresses modulate a sophisticated microbial ecosystem. Annu. Rev. Microbiol. 63, 431–450. 10.1146/annurev.micro.091208.073349 19575564

[B19] Hammes F. Verstraete W. (2002). Key roles of pH and calcium metabolism in microbial carbonate precipitation. Rev. Environ. Sci. Biotechnol. 1, 3–7. 10.1023/A:1015135629155

[B20] Hazen R. M. Papineau D. Bleeker W. Downs R. T. Ferry J. M. McCoy T. J. (2008). Review Paper. Mineral evolution. Am. Mineral. 93, 1693–1720. 10.2138/am.2008.2955

[B21] Holtz L.-M. Langer G. Rokitta S. D. Thoms S. (2013). Synthesis of nanostructured calcite particles in coccolithophores, unicellular algae, in Green Biosynthesis of Nanoparticles - Mechanisms and Applications, eds Rai M. Posten C. (Boston, MA: CABI Press), 132–147. 10.1079/9781780642239.0132

[B22] Hou W. Lian B. Zhang X. (2011). CO_2_ mineralization induced by fungal nitrate assimilation. Bioresour. Technol. 102, 1562–1566. 10.1016/j.biortech.2010.08.080 20880701

[B23] Ionescu D. Spitzer S. Reimer A. Schneider D. Daniel R. Reitner J.. (2014). Calcium dynamics in microbialite-forming exopolymer-rich mats on the atoll of Kiritimati, Republic of Kiribati, Central Pacific. Geobiology 13, 170–180. 10.1111/gbi.12120 25515845

[B24] Kang I. M. Roh K. M. (2013). Mineral carbonation of gaseous carbon dioxide using a clay-hosted cation exchange reaction. Environ. Technol. 34, 3191–3195. 10.1080/09593330.2013.821140 24617079

[B25] Kenward P. A. Fowle D. A. Goldstein R. H. Ueshima M. González L. A. Roberts J. A. (2013). Ordered low-temperature dolomite mediated by carboxyl-group density of microbial cell walls. Am. Assoc. Pet. Geol. Bull. 97, 2113–2125. 10.1306/05171312168

[B26] Kim O. S. Cho Y. J. Lee K. Yoon S. H. Kim M. Na H.. (2012). Introducing EzTaxon-e: a prokaryotic 16S rRNA gene sequence database with phylotypes that represent uncultured species. Int. J. Syst. Evol. Microbiol. 62, 716–721. 10.1099/ijs.0.038075-0 22140171

[B27] Kröger R. (2015). Biomineralization: ion binding and nucleation. Nat. Mater. 14, 369–370. 10.1038/nmat4256 25801404

[B28] Lee J. Y. Kim C. G. Mahanty B. (2014). Mineralization of gaseous CO_2_ by Bacillus megaterium in close environment system. Water Air Soil Pollut. 225, 1–8. 10.1007/s11270-013-1787-7

[B29] Lian B. Chen Y. Tang Y. (2010). Microbes on carbonate rocks and pedogenesis in Karst regions. J. Earth Sci. 21, 293–296. 10.1007/s12583-010-0240-8

[B30] Lian B. Hu Q. Chen J. Ji J. Teng H. H. (2006). Carbonate biomineralization induced by soil bacterium *Bacillus megaterium*. Geochim. Cosmochim. Acta 70, 5522–5535. 10.1016/j.gca.2006.08.044

[B31] Liu L. Zhang X. Liu X. Liu J. Lu G. Kaplan D. L.. (2015). Biomineralization of stable and monodisperse vaterite microspheres using silk nanoparticles. ACS Appl. Mater. Interfaces 7, 1735–1745. 10.1021/am507309t 25578091

[B32] Lowenstam H. A. Weiner S. (1989). On Biomineralization. New York, NY: Oxford University Press.

[B33] Mann K. Siedler F. Treccani L. Heinemann F. Fritz M. (2007). Perlinhibin, a cysteine-, histidine-, and arginine-rich miniprotein from abalone (*Haliotis laevigata*) nacre, inhibits *in vitro* calcium carbonate crystallization. Biophys. J. 93, 1246–1254. 10.1529/biophysj.106.100636 17496038 PMC1929040

[B34] Obst M. Dynes J. J. Lawrence J. R. Swerhone G. D. W. Benzerara K. Karunakaran C. (2009a). Precipitation of amorphous CaCO_3_ (aragonite-like) by cyanobacteria: a STXM study of the influence of EPS on the nucleation process. Geochim. Cosmochim. Acta 73, 4180–4198. 10.1016/j.gca.2009.04.013

[B35] Obst M. Wehrli B. Dittrich M. (2009b). CaCO_3_ nucleation by cyanobacteria: laboratory evidence for a passive, surface−induced mechanism. Geobiology 7, 324–347. 10.1111/j.1472-4669.2009.00200.x 19476505

[B36] Qin S. Li J. Chen H. H. Zhao G. Z. Zhu W. Y. Jiang C. L.. (2009). Isolation, diversity, and antimicrobial activity of rare actinobacteria from medicinal plants of tropical rain forests in Xishuangbanna, China. Appl. Environ. Microbiol. 75, 6176–6186. 10.1128/AEM.01034-09 19648362 PMC2753051

[B37] Rautaray D. Ahmad A. Sastry M. (2004). Biological synthesis of metal carbonate minerals using fungi and actinomycetes. J. Mater. Chem. 14, 2333–2340. 10.1039/b401431f

[B38] Rivadeneyra M. A. Delgado G. Ramos-Cormenzana A. Delgado R. (1998). Biomineralization of carbonates by *Halomonas eurihalina* in solid and liquid media with different salinities: crystal formation sequence. Res. Microbiol. 149, 277–287. 10.1016/S0923-2508(98)80303-3 9766229

[B39] Rodriguez-Navarro C. Jimenez-Lopez C. Rodriguez-Navarro A. Gonzalez-Muñoz M. T. Rodriguez-Gallego M. (2007). Bacterially mediated mineralization of vaterite. Geochim. Cosmochim. Acta 71, 1197–1213. 10.1016/j.gca.2006.11.031

[B40] Rodriguez-Navarro C. Jroundi F. Schiro M. Ruiz-Agudo E. González-Muñoz M. T. (2012). Influence of substrate mineralogy on bacterial mineralization of calcium carbonate: implications for stone conservation. Appl. Environ. Microbiol. 78, 4017–4029. 10.1128/AEM.07044-11 22447589 PMC3346411

[B41] Saghaï A. Zivanovic Y. Zeyen N. Moreira D. Benzerara K. Deschamps P.. (2015). Metagenome-based diversity analyses suggest a significant contribution of non-cyanobacterial lineages to carbonate precipitation in modern microbialites. Front. Microbiol. 6:797. 10.3389/fmicb.2015.00797 26300865 PMC4525015

[B42] Saitou N. Nei M. (1987). The neighbor-joining method: a new method for reconstructing phylogenetic trees. Mol. Biol. Evol. 4, 406–425. 3447015 10.1093/oxfordjournals.molbev.a040454

[B43] Schultze-Lam S. Fortin D. Davis B. S. Beveridge T. J. (1996). Mineralization of bacterial surfaces. Chem. Geol. 132, 171–181. 10.1016/S0009-2541(96)00053-8

[B44] Shirling E. Gottlieb D. (1966). Methods for characterization of Streptomyces species. Int. J. Syst. Bacteriol. 16, 313–340. 10.1099/00207713-16-3-313

[B45] Srivastava S. Bharti R. K. Thakur I. S. (2015). Characterization of bacteria isolated from palaeoproterozoic metasediments for sequestration of carbon dioxide and formation of calcium carbonate. Environ. Sci. Pollut. Res. 22, 1499–1511. 10.1007/s11356-014-3442-2 25163561

[B46] Tang Y. Lian B. Dong H. Liu D. Hou W. (2012). Endolithic bacterial communities in dolomite and limestone rocks from the Nanjiang Canyon in Guizhou karst area (China). Geomicrobiol. J. 29, 213–225. 10.1080/01490451.2011.558560 22571668

[B47] Torres A. R. Martinez-Toledo M. V. Gonzalez-Martinez A. Gonzalez-Lopez J. Martin-Ramos D. Rivadeneyra M. A. (2013). Precipitation of carbonates by bacteria isolated from wastewater samples collected in a conventional wastewater treatment plant. Int. J. Environ. Sci. Technol. 10, 141–150. 10.1007/s13762-012-0084-0

[B48] Tourney J. Ngwenya B. T. (2009). Bacterial extracellular polymeric substances (EPS) mediate CaCO_3_ morphology and polymorphism. Chem. Geol. 262, 138–146. 10.1016/j.chemgeo.2009.01.006

[B49] Tourney J. Ngwenya B. T. (2014). The role of bacterial extracellular polymeric substances in geomicrobiology. Chem. Geol. 386, 115–132. 10.1016/j.chemgeo.2014.08.011

[B50] Uma V. S. Dineshbabu G. Subramanian G. Uma L. Prabaharan D. (2014). Biocalcification mediated remediation of calcium rich ossein effluent by filamentous marine cyanobacteria. J. Bioremed. Biodeg. 5, 257 10.4172/2155-6199.1000257

[B51] Wei Z. Liang X. Pendlowski H. Hillier S. Suntornvongsagul K. Sihanonth P.. (2013). Fungal biotransformation of zinc silicate and sulfide mineral ores. Environ. Microbiol. 15, 2173–2186. 10.1111/1462-2920.12089 23419112

[B52] Weiner S. Dove P. M. (2003). An overview of biomineralization processes and the problem of the vital effect. Rev. Mineral. Geochem. 54, 1–29. 10.2113/0540001

[B53] Xiao L. Lian B. Hao J. Liu C. Wang S. (2015). Effect of carbonic anhydrase on silicate weathering and carbonate formation at present day CO_2_ concentrations compared to primordial values. Sci. Rep. 5, 7733. 10.1038/srep07733 25583135 PMC4291579

